# Annexins in Translational Research: Hidden Treasures to Be Found

**DOI:** 10.3390/ijms19061781

**Published:** 2018-06-15

**Authors:** Sebastian Schloer, Denise Pajonczyk, Ursula Rescher

**Affiliations:** Institute of Medical Biochemistry, Centre for Molecular Biology of Inflammation, University of Muenster, Von-Esmarch-Street 56, D-48149 Muenster, Germany; sebastschl@gmx.de (S.S.); Denise.Pajonczyk@ukmuenster.de (D.P.)

**Keywords:** annexins, inflammation, host-pathogen interplay, drug target, translational research

## Abstract

The vertebrate annexin superfamily (AnxA) consists of 12 members of a calcium (Ca^2+^) and phospholipid binding protein family which share a high structural homology. In keeping with this hallmark feature, annexins have been implicated in the Ca^2+^-controlled regulation of a broad range of membrane events. In this review, we identify and discuss several themes of annexin actions that hold a potential therapeutic value, namely, the regulation of the immune response and the control of tissue homeostasis, and that repeatedly surface in the annexin activity profile. Our aim is to identify and discuss those annexin properties which might be exploited from a translational science and specifically, a clinical point of view.

## 1. Introduction

Annexins stepped into the light in 1978, when a soluble protein was isolated from bovine adrenal glands that caused the aggregation of secretory vesicles in vitro when free Ca^2+^ was present [1]. This protein, initially called “synexin”, turned out to be the first discovered member of a new protein family, the annexins [2]. The Ca^2+^-dependent binding to phospholipid-containing membranes turned out to be their hallmark, mediated by their signature feature, the annexin repeat. More than 500 different members of the superfamily have now been identified [3]. According to the official nomenclature proposed in 1999, the 12 annexins commonly found in vertebrates constitute the A subclass [4]. Structurally, annexins all share the characteristic and highly conserved “core” domain, usually made up of four annexin repeats, each of which typically contains a Ca^2+^-binding motif and mediates the specific binding to negatively charged phospholipids. In 1990, the first crystal structure (of AnxA5) confirmed the predictions [5]. The tightly packed and slightly convex annexin core domain is linked to an N-terminal part (sometimes also called the “head” or “tail” domain) that is unique for a given annexin. N-terminal tails are surprisingly diverse in length and sequence, and sometimes contain binding sites for interaction partners, including members of the S100 family of EF-hand-containing Ca^2+^-binding proteins [6]. In several annexins, the tail is a substrate for kinases that have a strong influence on a wide variety of signal pathways, such as the proto-oncogene tyrosine-kinase Src and the Ca^2+^-controlled serine-threonine kinase PKC [7,8,9]. Phosphorylation is thought to regulate the protein function [5,10] and has been reported to control secretion, at least in the case of AnxA1 and A2, through a yet unknown unconventional pathway of these otherwise cytosolic proteins [11,12,13,14,15]. Not surprisingly, annexins have been implicated in the regulation of a broad range of cellular and physiological processes that are linked to cellular membranes, such as vesicle organization, membrane trafficking and scaffolding, endo- and exocytosis, and membrane/cytoskeleton interactions [16,17,18,19,20,21]. Membrane dynamics is also a recurrent theme in host–pathogen interactions, and annexins might function as host cell-derived auxiliary proteins in shaping the microbe–host interplay [22]. In recent years, a growing number of annexin knock out (KO) mouse models have been constructed [23], and they will certainly prove to be useful tools for investigating annexin functions, both as drugs and therapeutic targets.

## 2. Extracellular Functions—Detection of Phosphatidylserine and Immuno-Evasion

During pathophysiological responses, typical changes in the membrane composition and loss of membrane asymmetry are repeatedly observed. A prominent feature is the translocation of phosphatidylserine (PS), which in viable cells is located in the cytosol-facing leaflet of the plasma membrane, to the outside-facing leaflet of the apoptotic cell membrane [24]. In the presence of Ca^2+^, PS is a high-affinity ligand for the annexins and Ca^2+^-dependent PS binding is in fact, a defining trait of the annexin family [25]. For AnxA5, a *K_D_* value of 5 × 10^−10^ in the presence of Ca^2+^ [26] underscores the high selectivity in its preference for PS over other negatively charged phospholipids, and this specificity is the reason behind the wide use of labelled AnxA5 for the identification of apoptotic cells [27], for example in flow cytometry applications [28,29].

The surface-exposed PS assists in the recognition and subsequent phagocytic engulfment, of dying cells [30]. This process is called efferocytosis and is immune-calming in its nature [30] and seems to depend on the concomitant externalization of AnxA1 [31], which is part of the apoptotic cell-associated molecular patterns (ACAMPs) [32] that is presented by dying cells and conveys the switch towards an anti-inflammatory response. In accordance with the function as an “eat-me” signal, which most likely includes the acquisition of PS-bound anxA1 on the outer surface, phagocytosis of PS-decorated red blood cells is inhibited when PS is masked [33], for example through PS-binding proteins. Recent findings suggest that exposition of PS on the outer leaflet is not confined to apoptosis but appears to act as an evolutionary conserved global immunosuppressive signal [34], and is also found on the surface of cancer cells [35]. Unfortunately, in this context, PS exposure is not linked to cell elimination but seems to function in immune evasion [34], which, like in apoptotic cells, might depend on cell surface associated AnxA1 [36]. Blocking of PS with AnxA5 might be a strategy to antagonize the immune-suppression and help establish an anti-tumor immune reaction. Furthermore, AnxA5 might be used for the development of selective molecular imaging probes for cancer diagnosis and disease management [29,37] and importantly, for targeting drugs to the cancer cells [25,35].

## 3. Extracellular Functions—Annexins and Coagulation

Exposure of PS is also an important step in the regulation of blood clotting [38]. PS on the surface of endothelial cells or membrane vesicles derived from activated platelets greatly enhances the pro-thrombin/thrombin conversion which is a central unit in coagulation [39]. Annexin A5 is abundantly found on the surface of the syncytiotrophoblast, which covers the placental surface, and the AnxA5 layer is considered to protect the placenta from abnormal coagulation [40]. Furthermore, a polymorphism in the *AnxA5* gene was found to be associated with recurrent pregnancy loss. Women with the SNP in the *AnxA5* gene had a significantly higher risk of fetal loss than non-carriers [41]. The AnxA5 anticoagulant function might depend on its well-established property to self-assemble on PS-containing membranes into an extensive two-dimensional crystal lattice [42] that hinders the assembly of the pro-coagulant complexes. In line with such protective function in the blood clotting regulation, anti-AnxA5 autoantibodies are found in patients suffering from anti-phospholipid syndrome [43], a disease that manifests clinically as recurrent thrombotic events and is associated with fetal loss [44]. The occurrence of AnxA5 autoantibodies is also linked to autoimmune disorders [45,46] as observed in some patients suffering from multiple sclerosis or systemic lupus erythematosus. The current therapeutic strategy is long-term oral anticoagulation. Here, AnxA5 could be used to selectively neutralize the pathologic AnxA5 autoantibodies in vivo.

Among the many functions exerted by thrombin is the conversion of fibrinogen to fibrin which, together with platelets, forms a stable haemostatic plug that seals the injured vessel wall. To avoid excessive clot formation, the damaged endothelium slowly secrets components that assist in the conversion of plasminogen entrapped in the clot to enzymatically active plasmin, which breaks down the fibrin mesh. AnxA2, possibly as a heterotetramer together with its ligand S100A10, was demonstrated to enhance plasmin generation [47,48]. Consistently, AnxA2 KO mice present defective fibrinolysis and increased thrombotic vascular occlusion and impaired neovascularization [49]. Blast cells of patients with acute promyelocytic leukemia (APL) express AnxA2 to a high amount [50], which might explain the haemorrhagic complications observed in APL patients. In line with the impact of AnxA2 on coagulopathy [50], treatment with the retinoic acid receptor ligand, all-trans retinoic acid (ATRA), attenuates AnxA2 expression and improves clinical resolution. Thus, annexin-based biologicals might serve as novel agents that open new therapeutic options and might be part of the management of such diseases in the future.

## 4. Extracellular Functions—Annexins as Ligands of Defined Inflammation-Related Receptors

A conceptually straightforward approach is to therapeutically exploit those annexins which function as endogenous ligands for known receptors. In this regard, the most prominent annexin is certainly AnxA1. Still under its former name lipocortin 1, AnxA1 gained considerable interest as a key mediator of glucocorticoid actions in inflammation. AnxA1 deficient mice do not respond to glucocorticoid treatment under inflammatory conditions [51]. The full-length protein as well as its famous Ac2-26 N-terminal peptide pharmacophore, which might be proteolytically released from the full length protein [32,52], act in an anti-inflammatory manner in many experimental conditions, and we refer the reader to the many excellent and comprehensive review articles on that topic [53,54,55,56]. A molecular explanation was provided by the discovery that both AnxA1 and the Ac2-26 peptide specifically bind and activate the formyl-peptide receptor (FPR) subfamily [57,58] of heptahelical, G-protein coupled cell surface receptors. Most of the reported AnxA1 anti-inflammatory functions depend on binding to FPRs. However, it is entirely possible that additional signaling mechanisms elicited through yet unknown signalling receptors are involved, as the recognition of an annexin core (which can also be derived from annexins other than A1) also contributes to an immune-modulation [59]. In humans, three members of the FPR family are found: FPR1, FPR2, and FPR3, whereas in mice at least eight FPRs are expressed [60]. The most prominent receptors among the FPR family expressed in the murine model are FPR1 and FPR2 [61]. FPR1 and FPR2 are predominantly expressed on the surface of many immune cells (e.g., neutrophils, macrophages, dendritic cells) but also found in endo- and epithelial cells [62]. A broad range of FPR1 ligands, both agonist and antagonists, have been described [63], and autocrine/paracrine signalling of externalized AnxA1 protein and/or its peptides via the FPRs might explain its well-known immune-modulatory and pro-resolving actions. Upregulation of AnxA1 expression is observed in several inflammatory conditions [64] and thought to function in resolution and tissue protection [65]. Indeed, studies on the use of Ac2-26-containing nanocapsules in the treatment of mucosal injury in the murine model, reported enhanced colonic wound healing, both in the acute and chronic situation [66]. Interestingly, a small peptide derived from the AnxA1 N-terminus attenuated experimental colitis in mice [67]. Chronic inflammation is also observed in obesity [68]. Interestingly, AnxA1 KO mice on a high-fat diet are more prone to obesity than the control animals [69], and FPR2 activation improved systemic insulin sensitivity [70].

The AnxA1/FPR signaling axis might constitute an attractive target for the treatment of cardiovascular diseases. We only give a cursory overview as we want to draw the reader’s attention to the excellent review articles included in this special issue “Annexins—Closing the Gap between Fundamental and Translational Research”. A lowered AnxA1 expression in plaques obtained from patients with carotid stenosis correlates with neurological symptoms [71]. Correspondingly, vulnerable plaque regions obtained from human carotid endarterectomy were shown to have less pro-resolving factors, such as resolvin D1 (RvD1), compared to more stable regions [72]. RvD1 is another ligand for FPR2 [73], thus indicating a potential therapeutic use of AnxA1 to support the resolution phase, suppress plaque progression, and enhance plaque stability. A growing body of evidence also points at an anti-inflammatory and neuroprotective function of AnxA1 in the brain, and AnxA1-derived molecules might emerge as promising tools in the treatment of brain diseases, including stroke and neurodegenerative disorders [74,75]. The decisive role of AnxA1 in stroke development and progression has been highlighted in a global murine stroke model mimicking cerebral ischemia caused by atherosclerosis, which is accompanied by cardiac arrest. Ischemic mice treated with AnxA1or the Ac2-26 peptide presented reduced infarct size, less cerebral edema, and improved neurological score [76]. Furthermore, Ac2-26 prevents neutrophil-platelet aggregate formation within cerebral microvessels through the interaction with FPR2 [77].

A caveat to the generalized use of AnxA1 for treatment of excess inflammatory conditions is the observation that LPS, a highly potent pro-inflammatory component derived from the bacterial wall of gram-negative bacteria, triggers the upregulation of AnxA1 expression in a variety of cell types e.g., neutrophils [78]. Indeed, elevated AnxA1 plasma levels are found in 56% of septic patients after hospital admission [79]. While initially beneficial [78], it remains to be investigated whether excess LPS-induced AnxA1 externalization might cause the so-called endotoxin resistance, a dangerous refractive state of the innate immune system characterized by a lowered response towards a second exposure to bacterial lipopolysaccharide [80]. However, the target delivery of AnxA1-derived compounds has tremendous promise to treat a range of inflammatory conditions.

AnxA2 not only impacts fibrinolysis but (in its heterotetrameric form together with S100A10) affects the Toll-like receptor (TLR) signaling. The AnxA2-S100A10 complex activates human and murine macrophages through the TLR4-MyD88 pathway, although the cell’s responsiveness requires an additional and yet unknown factor [81,82,83]. Signaling through the TRAM/TRIF-module of the TLR4 pathway was reported to attenuate Klebsiella-induced lung inflammation in a murine model of acute pneumonia [83]. Monomeric AnxA2 was also shown to bind to and activate TLR2 via its N-terminal domain, thus assisting in the differentiation of antigen-presenting cells [84]. Extracellular AnxA2 was shown to also interact with the proprotein convertase subtilisin/kexin-type 9 (PCSK9), thus interfering with PCSK9-mediated degradation of the hepatic low-density lipoprotein receptor (LDLR) [85,86,87]. Because PCSK9 reduces the number of LDL receptors on the liver cell membrane, PCSK9 inhibitors can be used to increase the number of cell surface LDL receptors and thereby reduce cholesterol levels in the blood. Here, AnxA2 might be used as a PCSK9 inhibitor to treat hypercholesterolaemia.

In search of the molecular mechanism underlying the stimulatory effects of AnxA2 on human osteoclast formation [88,89], a novel type I membrane protein was identified as a putative AnxA2 receptor [90]. A recent study linked a single nucleotide polymorphism (SNP) in the *AnxA2* gene (rs7170178) to osteonecrosis in sickle cell patients. The SNP frequency of the *AnxA2* gene polymorphism was higher in sickle cell osteonecrosis patients than those without osteonecrosis [91]. Interaction of AnxA2 with the AnxA2 receptor also mediates adhesion and activation of the cells responsible for the initiation and maintenance of multiple myeloma [92] and this signal pathway could be used as a therapeutic target.

## 5. Intracellular Functions in Pathophysiological Scenarios

The following sections will cover those intracellular functions of annexins which are of potential relevance in pathophysiological scenarios. In the simplest approach, characteristic changes in the annexin expression profile might be used to diagnose and monitor diseases and to predict and evaluate therapeutic success. It is entirely possible that the relative expression levels of several annexins constitute valuable panels of biomarkers, and that such an annexin-based multibiomarker-model could be used to estimate the disease risk. Approaches addressing intracellular proteins are challenging and require sophisticated technologies that are only beginning to emerge. In this case, annexins might be used for gene and cell therapy approaches or might serve as druggable targets for cell-penetrating small molecules that interfere with or mimic annexin functions. Additionally, the intracellular delivery of annexin-derived therapeutics (e.g., as cell-penetrating fusion peptides) might be exploited in intracellular protein therapy.

### 5.1. Intracellular functions—Annexins as Biomarkers

**Figure 1:**
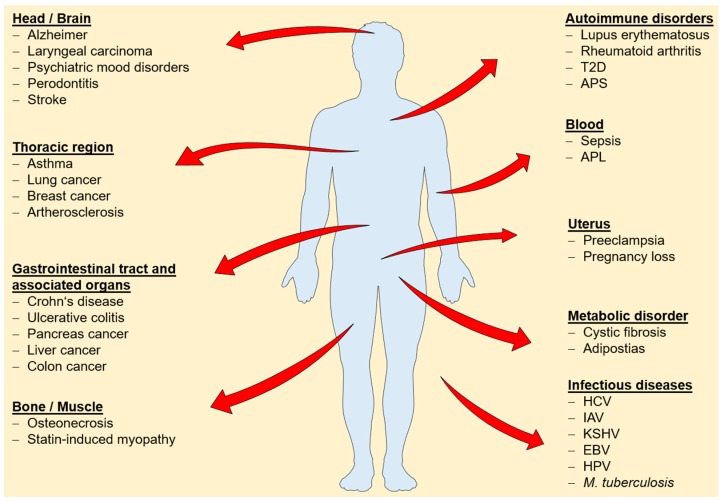
Overview of diseases associated with changes in annexin expression levels. Abbreviation: T2D: Type 2 diabetes mellitus [93]; APS: antiphospholipid syndrome [43]; APL: promyelocytic leukemia [50]; HCV: hepatitis C virus [94]; IAV: influenza A virus [95]; KSHV: Kaposi’s sarcoma-associated herpesvirus [96]; EBV: Epstein-Barr-Virus [97]; HPV: *Human papillomavirus* [98]; *M. tuberculosis*: *Mycobacterium tuberculosis* [99].

Changes in cellular or tissue expression levels have been reported for several annexins and a broad range of diseases (Figure 1), suggesting a potential use to determine onset and progression of disease and to monitor therapeutic success. The following studies are exemplary only and illustrate the potential use of determining annexin expression profiles for the early detection of common cancers.

Massive dysregulation of annexin expression patterns occur during tumorigenesis. For instance, serum levels of AnxA1 are significantly elevated in lung cancer patients. Due to the strong association of AnxA1 to the pathological grade and clinical stage, it is a convenient marker for monitoring the course of disease [100]. Furthermore, AnxA1 expression is upregulated in skeletal muscle of non-myopathic patients undergoing statin therapy, and was proposed to serve as a biomarker for T-tubular repair in those patients [101].

Annexin expression profiles are often changed in tumorous tissues. For instance, the AnxA2 expression is significantly associated with tumor size, lymph node metastasis, distant metastasis and clinical stage of laryngeal cancer, and therefore it is a promising candidate for estimating the prognosis of patients with laryngeal carcinoma or gliomas [102]. AnxA10 is already used as a biomarker for hepatocellular carcinoma (HCC) and is markedly downregulated during cancer progression [103]. The AnxA10 downregulation, together with a characteristic p53 mutation, acts synergistically toward high-grade, high-stage HCC and goes along with poorer prognosis [103]. AnxA10 might be useful to identify adenocarcinomas of unknown primary origin, as AnxA10 expression is commonly found in carcinoma of the upper gastrointestinal tract and the pancreatobiliary system [104].

### 5.2. Intracellular Functions—Regulation of Cytosolic Phospholipase A2 (cPLA2) Enzymatic Activity

Very early on, the ability of annexins, and especially AnxA1 and AnxA2, to inhibit cPLA2, thus interfering with arachidonic acid release and eicosanoid formation, has been acknowledged. Mechanistically, the function was explained by competition for the lipid substrates [105,106,107]. However, cPLA2 might be inhibited in a more direct manner [108]. Given the fundamental role of this enzymatic activity in eicosanoid production, annexins might serve as a starting point to discover new lead structures for further cPLA2 inhibitors.

### 5.3. Intracellular Functions—Cell Surface Presentation of Integral Plasma Membrane Molecules

The AnxA2-S100A10 tetramer has been shown to interact with (and possibly regulate) a number of integral plasma membrane molecules including ion channels and receptors, like the Ca^2+^-selective Transient Receptor Potential vanilloid type 5 and 6 channels (TRPV5 and TRPV6) [109], the acid-sensing ion channel ASIC [110], the two-pore-domain potassium channel TASK-1 [111], the chloride channel Cystic Fibrosis Transmembrane Conductance Regulator (CFTR) [112] the GPCR CCR10 [113] and the 5-HT1B receptor [114]. Interfering with these S100A10 interactions could be envisioned for the treatment of the corresponding diseases, including depression [114], although a beneficial effect of an upregulation of S100A10 expression, e.g., through 1,25-dihydroxyvitamin D3 [109], still needs to be demonstrated.

In many cases, altered annexin expression correlates with changes in migratory behaviour and invasiveness [115,116,117,118,119,120], and an altered cell surface environment might be the underlying cause. For instance, regulatory functions in the presentation of cell adhesion proteins, such as integrins, have been elegantly demonstrated for AnxA6 and AnxA8 [115,116]. Because these cell surface receptors cluster at focal contacts to form effective cell-cell or cell-matrix contacts, the number of integrins at the cell surface is a critical determinant of a cell’s motility. Cell adhesion and movement, in turn, is fundamental to cancer cell metastasis, wound healing, and angiogenesis. In this context, the pattern of annexin gene expression may correlate to physiological and pathophysiological wound healing phenotypes. Such information would assist in assessing the course of healing within a wound, and in identifying target pathways for appropriate treatment regimes.

### 5.4. Intracellular Functions—Plasma Membrane Repair

Very recently, another important annexin function related to cellular processes controlling damage appeared. To maintain a functional plasma membrane (PM), eukaryotic cells are able to repair PM injuries. The resealing is Ca^2+^-dependent and depends on a complex machinery. It can probably occur through different mechanisms, depending on the kind and extent of injury. PM repair is essential for skeletal muscle homeostasis, and defective PM repair manifests very impressively in skeletal muscle damage and is linked to degenerative muscle diseases such as myopathies and muscular dystrophies [121]. A growing body of research suggests that several annexin family members facilitate the required membrane fusion events during the healing of PM lesions [122,123,124,125,126,127,128]. Here, gene therapy, i.e., the transfer of DNA encoding functional annexin proteins into the target cells, might be used to treat conditions caused by defective PM repair mechanisms.

## 6. Annexins and the Host/Pathogen Interface

The growing appearance of antibiotic resistance is one of the major threats to human health. To overcome the emergence of drug-resistant viruses, bacteria, and fungi, therapeutic strategies that aim at targeting host cell factors rather than the pathogen itself are currently being pursued [129]. However, most of these novel approaches are still in the very early phases of clinical trials. For the development of such novel approaches, detailed knowledge of the manifold host/pathogen interactions that take place during the course of infection, is paramount. An obvious target for interventions is the host innate immune response. It acts as a first line of defense against pathogen attack, and, not surprisingly, pathogens have evolved sophisticated strategies to overcome these cellular defense mechanisms or even use them to their advantage. Trypanosoma cruzi, for example, presents PS on the cell surface of trypomastigote stage in order to mimic the anti-inflammatory effects of apoptotic cells, thus evading the host innate immune response [130]. A similar mechanism of apoptotic mimicry to balance inflammation is also used by *Toxoplasma gondii* [131] and *Leishmania braziliensis* [132], although it remains to be studied whether the immunomodulatory function also depends on the recruitment of AnxA1. However, blocking of the surface-exposed PS by AnxA5 impacts infectivity of *Toxoplasma* [131] and *Leishmania* [132]. These exemplary observations underscore that the elucidation of a functional role for the annexins during microbial infection, an emerging and rapidly growing field within the annexin research, holds potential for developing annexin-based therapeutic options.

Several annexins are incorporated into virus particles, for example, influenza A virus (IAV) particles contain annexins A1, A2, A4, A5, and A11 [133]. In addition to IAV, AnxA1 is found associated with several other viruses [133,134,135,136,137,138,139,140,141]. Interestingly, no indications so far suggest that the viruses rely on its immune-modulating capacity to facilitate virus entry, although the AnxA1 receptor FPR2 is involved in IAV replication. Notably, FPR2 activation increases viral replication, and FPR2 antagonists protect mice from lethal IAV infections [142,143]. A clearer active role during viral infection is found for AnxA2 and AnxA5. An exploitation of virus-incorporated AnxA2 to promote the conversion of plasminogen to plasmin on the cell surface was reported to be utilized, at least in a supportive manner, by herpesvirus and IAV [144,145,146,147]. Virus-incorporated AnxA5 assists IAV infection through the inhibition of interferon-mediated host cell protection [148]. Whether the specific binding of anxA5 to a hepatitis B virus (HBV) surface antigen [149,150] mediates a similar function, thereby affecting the host cell susceptibility to HBV [151,152,153,154] remains to be investigated. Although the potential importance of the remaining IAV-associated annexins is still unknown, the above indications point to a function, at least for several annexins, as host-derived virulence factors. Furthermore, the most common high-risk human papillomavirus HPV16, which causes benign and malignant mucosal and cutaneous epithelial tumors, induces and utilizes the AnxA2/S100A10 heterotetramer on the host cell surface for effective internalization. The complex most likely interacts with the HPV minor capsid protein L2 [155,156]. Importantly, interfering with AnxA2/S100A10 complex through the use of small molecule inhibitors markedly impairs HPV16 infection in a cell culture model [157]. Because a similar function for AnxA2 as a host cell receptor was also reported for several other viruses, including human cytomegalovirus, enterovirus type 71, rabbit vesivirus, and respiratory syncytial virus [158,159,160,161,162,163], as well as for *Pseudomonas aeruginosa* and *Mycoplasma* [164,165,166], such small molecule inhibitors might be of therapeutic value in a broad range of viral and bacterial infections. Further host-directed drug therapies might include targeting of other annexin/pathogen interactions, for instance through the use of synthetic mimetic peptides that effectively compete for interaction sites.

Because of their biochemical signature, i.e., the ability to dynamically bind to membrane phospholipids (and for some annexins, to F-actin) in response to fluctuating intracellular Ca^2+^ concentrations, annexins are perfectly suited to transduce and integrate membrane-related events and signaling. While there is a vast body of literature on their participation in e.g., endo- and exocytosis, membrane rearrangement events and cytoskeletal organization, growing evidence suggests that the establishment and control of membranes under non-equilibrium conditions is where the full annexin potential is unlocked. A predominant function of these proteins in the assembly of transient membrane domains and the maintenance of membrane integrity under potentially harmful conditions, such as PM disruption (see above) is also in accordance with the fact that annexin KO mice are viable, develop normally and have no evident phenotypic alterations during their lifetime [23].

Because microbes in the human body intimately associate with the membrane system of their host cells, they have evolved sophisticated strategies to hijack the cell machinery and use it to their advantage [167]. For instance, many invading pathogens need to penetrate the PM or endosomal membranes to get access to the cell interior in order to proliferate. These pathogen-induced membrane manipulations are potentially harmful, and a clear example is the insertion of pore-forming toxins, such as streptolysin-O (SLO), the pore-forming toxin of Streptococcus pyogenes. The host cell perceives the concomitant rise in intracellular Ca^2+^ as a danger signal indicating PM injury, a situation that closely resembles PM damage induced by mechanical forces. In line with their proposed function as part of the repair machinery that kicks in when a high Ca^2+^ influx is sensed [20,122], several annexins are involved in repairing the PM lesions caused by SLO [168,169]. This annexin-mediated resealing, blebbing, and subsequent shedding of the affected PM domains as pore-containing microvesicles is cell-protective and thus might constitute a vital part of the host cell innate immune response. The importance of the rapid and effective toxin removal for the host protection has been impressively demonstrated in vivo, as the administration of artificial liposomes that effectively compete with the host cell membranes sequester such toxins and successfully prevent the development of severe sepsis in a murine staphylococcal sepsis model [170]. Another example of a pathogen exploiting the host cell PM repair mechanism is the induction of lysosomal exocytosis during the host cell entry process of adenoviruses [171]. Fusion of lysosomes with the PM is also a means to control the propagation of intracellular bacteria [172]. As annexins are involved in at least certain lysosomal fusion events [173,174], it is highly likely that they also play an important part in these pathogen-related events.

In many cases, annexin-mediated reorganization of intracellular membranes affects the pathogen propagation. IAV infection, for instance, is significantly reduced when AnxA6 is overexpressed, causing imbalanced cholesterol levels in the PM and endosomal membranes [175]. AnxA2 and A3 are involved in the production of Hepatitis C virus (HCV) particles, presumably through supporting the establishment of the “membranous web”, the highly specialized and supposedly endoplasmatic reticulum (ER)-derived sites of HCV replication in infected cells [176,177,178] and affecting HCV maturation and egress [179].

These examples are certainly far from being complete, but were selected to highlight the potential therapeutic value of the annexins in the development of novel, host-centered approaches, either as lead substances for drug design or promising targets in pathogen–host cell interactions.

## 7. Conclusions

This review summarizes the potential clinical use of the annexins. While by no means exhaustive (we apologize to any colleague whose excellent work had to be excluded in the interest of space), we believe that the special issue “Annexins—Closing the Gap between Fundamental and Translational Research” is a collection of exemplary articles that helps bring together annexin-themed basic science and translational research which will inspire a fresh look and open up a whole new world where these proteins can be conquered.
